# Detecting and correcting partial errors: Evidence for efficient control without conscious access

**DOI:** 10.3758/s13415-013-0232-0

**Published:** 2013-12-18

**Authors:** N. Rochet, L. Spieser, L. Casini, T. Hasbroucq, B. Burle

**Affiliations:** Laboratoire de Neurosciences Cognitives, UMR 7291, Fédération de Recherche 3C, Aix-Marseille Université and CNRS, Case C, 3, Place Victor Hugo, 13331 Marseille, France

**Keywords:** Cognitive control, Error detection, Action awareness

## Abstract

Appropriate reactions to erroneous actions are essential to keeping behavior adaptive. Erring, however, is not an all-or-none process: electromyographic (EMG) recordings of the responding muscles have revealed that covert incorrect response activations (termed “partial errors”) occur on a proportion of overtly correct trials. The occurrence of such “partial errors” shows that incorrect response activations could be corrected online, before turning into overt errors. In the present study, we showed that, unlike overt errors, such “partial errors” are poorly consciously detected by participants, who could report only one third of their partial errors. Two parameters of the partial errors were found to predict detection: the surface of the incorrect EMG burst (larger for detected) and the correction time (between the incorrect and correct EMG onsets; longer for detected). These two parameters provided independent information. The correct(ive) responses associated with detected partial errors were larger than the “pure-correct” ones, and this increase was likely a consequence, rather than a cause, of the detection. The respective impacts of the two parameters predicting detection (incorrect surface and correction time), along with the underlying physiological processes subtending partial-error detection, are discussed.

Keeping an adaptive behavior requires the efficient detection of processing failures in order to promptly react to incorrect actions, allowing an individual to correct them and avoid new errors. In the laboratory, processing failures are mainly studied in so-called “reaction time” (RT) tasks, in which participants must issue a quick response to sensory stimulations. Since the pioneering works of Rabbitt and colleagues (Rabbitt, 1966; Rabbitt & Vyas, 1981), it has been well established that participants can correct their errors in more than 95 % of the cases. Correlatively, after an error participants tend to slow down their responses (Laming, 1968, 1979; Rabbitt, 1966), likely to avoid making a new error (Dutilh et al., 2012; see, however, Notebaert et al., 2009). Such *posterror slowing* has been shown to occur only after consciously detected errors (Endrass, Reuter, & Kathmann, 2007; Nieuwenhuis, Ridderinkhof, Blom, Band, & Kok, 2001), making an explicit link between conscious error detection and the recruitment of executive processes.

In such RT tasks, errors are traditionally defined in a binary fashion, on the basis of the overt behavior: The buttonpress is classified as being either correct or erroneous. However, analyses of response-related EMG activity have revealed that erring is not all or none: About 15 %–20 % of correct overt responses are preceded by an early, subthreshold EMG burst from the hand that is associated with the incorrect response (Burle, Possamaï, Vidal, Bonnet, & Hasbroucq, 2002; Eriksen, Coles, Morris, & O’Hara, 1985; Gratton, Coles, & Donchin, 1992; Smid, Mulder, & Mulder, 1990), which is called a “partial error.” In such trials, the incorrect response activation has been successfully suppressed, preventing an overt error (see Fig. 1a and the “Method” section for more details).

**Fig. 1:**
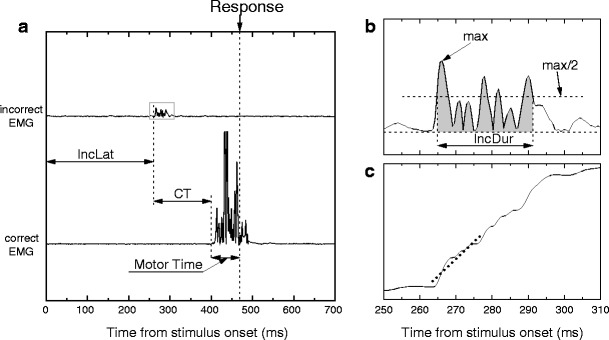
Example of a partial error, along with the extracted indices. **a** Typical electromyographic (EMG) recording showing a partial error. Time 0 is stimulus onset, and the long vertical dashed line indicates the mechanical response. The bottom trace presents the rectified EMG activity of the muscle involved in the correct response. A large EMG burst starts slightly before the mechanical response. This correct EMG burst is preceded by a small burst on the incorrect muscle (top trace), which is far too small to produce an overt response. The extracted indices are the latency of the partial error (*IncLat*), the correction time (*CT*, between the incorrect and the correct EMG burst onsets), and the *motor time* between the correct EMG burst onset and the mechanical response. **b** Zoom depiction of the partial error, depicting the extracted EMG burst parameters. First, we computed the maximum of the rectified trace. Then we extracted the earliest point preceding, and the latest point following, the peak whose amplitudes were equal to or larger than half of the max amplitude. The time separating the two values was taken as the measure of EMG burst duration (*IncDur* and *CorDur*, for incorrect and correct EMG bursts, respectively). The surface under the curve between these two points (shaded area in panel **b**) was taken as a measure of the EMG burst amplitude (*IncSurf* and *CorSurf*, for incorrect and correct bursts, respectively). **c** Slope extraction: The cumulative sum of the rectified EMG trace was computed, becoming monotonically increasing. The linear trend was then removed to get a “flat” signal. A linear regression was computed on the first 30 points of the cumulative signal following the burst onset (i.e., on about the first 15 ms), and the slope of the regression (dashed line in panel **c**) is taken as a measure of the steepness of the EMG burst (*IncSlope* and *CorSlope*, for incorrect and correct EMG bursts, respectively)

Trials containing a partial error are thus of particular interest, since they indicate that although an error was about to be made, the nervous system was able to overcome it and provide the correct action. In the occulomotor domain, it has been reported that not all incorrect eye movements are detected (Endrass et al., 2007; Nieuwenhuis et al., 2001; O’Connell et al., 2007). Whether incorrect response activations without movement can be detected is still an open question, since, to the best of our knowledge, nobody has yet addressed this issue. Indirect assessments have provided contrasting answers. First, overt errors are known to elicit two brain responses: early fronto-medial brain activity, known as the “error negativity” (Ne: Falkenstein, Hohnsbein, Hoormann, & Blanke, 1991; also termed the “error-related negativity,” or ERN: Gehring, Goss, Coles, Meyer, & Donchin, 1993), followed by a later positive deflection termed the “error positivity” (Pe: Falkenstein et al., 1991). It has been shown that the Ne is generated independently of the conscious detection of errors, whereas the Pe seems to be specific to detected errors (Endrass et al., 2007; Nieuwenhuis et al., 2001; O’Connell et al., 2007). Interestingly, partial errors elicit a clear Ne, but no Pe (Burle, Roger, Allain, Vidal, & Hasbroucq, 2008; Vidal, Hasbroucq, Grapperon, & Bonnet, 2000), suggesting that they remain largely undetected. On the other hand, it has been shown that cognitive adjustments do occur after partial errors, such as increased incorrect-response suppression (Burle et al., 2002) or post-partial-error slowing (although this is weaker than slowing after overt errors; Allain, Burle, Hasbroucq, & Vidal, 2009). Such data are indicative of control adjustments occurring after partial errors. Since it is often assumed that the recruitment of executive control processes requires conscious access (Dehaene & Naccache, 2001), this would indicate that partial errors are consciously detected. On the contrary, if they are not consciously detected, this would indicate that control mechanisms can occur without conscious access (van Gaal, Lamme, & Ridderinkhof, 2010).

Studying whether participants can consciously detect their partial errors was the first goal of this study. Because we anticipated that some, but not all, of the partial errors would be detected, the second goal was to search for the determinants of conscious (non)detection.

Toward this aim, we first analyzed chronometric and EMG indices to identify the differences between detected and undetected partial-error trials (see below for the detection methodology). We then investigated whether conscious detection could be predicted on the basis of those indices and the EMG signal, and if so, which parameters were the best predictors of conscious detection.

## Method

### Participants

A group of 18 right-handed participants with normal or corrected-to-normal vision (six men and 12 women; mean age 25.1 years, ranging from 19 to 38 years) volunteered for this study and were paid €10 per hour. All of the participants were neuroscience or psychology students (bachelor-level minimum), so that they knew what EMG was and could understand what a “partial error” meant. They all gave written informed consent for taking part in this study and were free of any psychological or neurological disorders. This experiment was approved by the ethics committee of Aix-Marseille University and by the Comité de Protection des Personnes Sud Méditerranée 1 (Approval No. 1041).

### Stimuli and procedure

Stimuli were delivered by three light emitting diodes (LEDs) presented on a panel placed 1 m in front of the participants. The central LED served as fixation point, and was symmetrically surrounded by two lateral LEDs, displaying the response signals. The visual angle between the fixation and the center of each lateral LED was 5.1º. A trial started when the central LED was turned on blue for 150 ms. Five hundred milliseconds later, one of the two lateral LEDs was lighted unpredictably either in green or red, to which participants had to respond with their left or right thumb by pressing one of two response buttons (left or right, separated by 20 cm) placed in front of them. The LED displaying the stimulus remained on until participant’s response or for 1,000 ms if no response was given. All of the stimuli and responses were controlled by a PC running a custom-made computer program based on Tscope (Stevens, Lammertyn, Verbruggen, & Vandierendonck, 2006).

Participants performed a Simon task (Simon, 1990) in which they had to respond as quickly and accurately as possible as a function of the color of the stimulus and to ignore the stimulus position. Responses were delivered by closing the response buttons (force necessary for closure: 7 N). When the stimulus and the response were ipsilateral, the trial was termed “congruent”; in the opposite case, it was termed “incongruent.” The color-to-response mapping was counterbalanced across participants and changed in every experimental session (see below) for each participant.

Concerning partial-error detection, they received the following written instructions (translated from French): “When you respond, you will have to try to feel if you have made a partial error—that is, if you feel you have produced EMG activity in the thumb muscle located on the side opposite the expected response.” A trace of a partial error was presented below this text, and was followed by the remainder of the instructions:

At the end of each trial, a graduated scale ranging from 1 to 6 will be displayed on the monitor screen located in front of you, and you will have to orally evaluate your certainty about having produced a partial error (from 1 if you are *sure you have not produced a partial error*, to 6 if you are *sure you have produced one*). If you think you have committed an error, say “Error.”

The experimenter typed the confidence level on the keyboard of the computer controlling the experiment, which turned off the confidence scale. The next trial started 1,500 ms after.

Each participant performed three sessions of 12 blocks of 64 trials (i.e., a total of 2,304 trials per participant). Prior to each session, participants performed a training block of 64 trials. Each second and third session was performed at least a day after the previous one. The color-to-response-side association was changed in every session, in order to increase the likelihood of partial errors. Half of the participants had to respond with the right thumb for a green stimulus and with the left thumb for a red stimulus on the first session, and used the opposite mapping for the second session. For the other half, the order was reversed. For one participant (#7), one session could not be analyzed because of artifacts in the EMG signal; her or his data are hence only based on two sessions.

### EMG recording

Participants were seated in a comfortable chair. The EMG activity of their flexor pollicis brevis of both hands was recorded with two surface active-two (Biosemi, Amsterdam, The Netherlands) Ag–AgCl electrodes glued approximately 2 cm apart on the thenar eminences. This activity was sampled at 2048 Hz (analog bandwidth limit: –3 dB at 1/5th of the sampling rate). The EMG signal was continuously monitored by the experimenter in order to avoid, as much as possible, any small background activity that could mask small muscles activations. In particular, if the EMG signal showed tonic muscular activity, the experimenter asked the participant to relax his or her muscles.

### Detection categories

For all of the analyses, trials rated with a confidence level of 1 or 2 were considered to be “undetected” partial errors whereas trials rated with a confidence level of 5 or 6 were considered to be “detected” partial errors. Trials rated as 3 and 4 were considered “uncertain.” In a first step, we concentrated on the “detected” and “undetected” categories in order to apply signal detection theory (SDT; Green & Swets, 1966) tools. According to SDT, trials were classified in four types: hits (correct report of a partial error), misses (no report of a partial error when there was one), false alarms (partial error reported when none was present), and correct rejections (no partial error reported when none was present). Second, we analyzed the effect of detection category (undetected, uncertain and detected) on the chronometric and physiological EMG parameters (see below).

### EMG signal processing

The EMG signal was high-pass-filtered offline at 10 Hz. EMG data were processed with BrainAnalyzer (BrainProducts, Munich, Germany) and with custom programs (written in MATLAB [The Mathworks, Natick, MA] or Python [www.python.org]). First, the onset and the offset (for partial errors) of each EMG burst was marked manually after visual inspection and trials containing artifacts were removed. Indeed, although automated algorithms can be useful, visual inspection remains the most accurate technique against which all algorithms are compared (van Boxtel, Geraats, van den Berg-Lessen, & Brunia, 1993). Note that the experimenter was not aware of the type of trial that he was marking. Trials were then classified as correct or erroneous, depending on whether the correct or incorrect response button was pressed first. Among the correct trials, we separated those containing one EMG burst on the correct side only (pure-correct trials) and trials containing an EMG burst on the incorrect side preceding the one on the correct side (partial-error trials). More precisely, a partial-error trial was defined as a behaviorally correct trial in which the correct EMG burst was preceded by EMG activity on the incorrect muscle that was insufficient to produce an overt response (see Fig. 1a).

For partial-error trials, nine parameters were extracted, to investigate how they related to conscious detection (see Fig. 1). First, we measured three chronometric parameters: the latency of the incorrect EMG burst (IncLat); the correction time (CT), defined as the time between the incorrect and correct EMG bursts onsets; and the motor time (MT) that separated the correct EMG onset from the mechanical response.

In addition, six EMG parameters were computed from the rectified EMG signal: the surface area under the incorrect (IncSurf) and correct (CorSurf) EMG bursts, the duration of the incorrect (IncDur) and correct (CorDur) EMG bursts, and the leading edges of the two EMG bursts (IncSlope and CorSlope for the incorrect and correct EMGs, respectively). To obtain more robust estimates of those parameters, they were computed as follows: The peak (i.e., the highest value of the EMG burst) was detected. The value corresponding to the half amplitude was then computed, and the first and last points of the rectified EMG burst that crossed the half value were determined (see Fig. 1b). The time between these two points was used as the duration of the burst (IncDur and CorDur, for incorrect and correct EMG bursts, respectively). The surface under the EMG curve within this interval was used as IncSurf and CorSurf, for incorrect and correct EMG bursts, respectively. These estimates are more robust than the raw ones (based on EMG onset and offset) because they are independent of EMG onset, and especially of the offset, which is difficult to determine.^1^ For the same reasons, to estimate the leading edge of the EMG burst, we first computed the cumulative sum of the EMG burst (which becomes monotonically increasing), and after detrending, we fitted a linear regression on the first 30 points (corresponding to about 15 ms) of this cumulative sum (see Fig. 1c). The slope of the regression was used as a measure of the steepness of the leading edge.

We then investigated which of those EMG parameters differed between undetected, uncertain, and detected partial-error trials. Toward this aim, for each parameter, we first computed a two-way analysis of variance (ANOVA) for repeated measures, with the factors Congruency and Detection.

Those parameters provide good summaries of the EMG shape but do not provide a complete shape of the EMG burst. To better assess the global shape, the rectified EMG bursts were averaged, time-locked to the EMG burst onset. From those averaged signals, three parameters were extracted for each participant and experimental condition: the amplitude (measured as the peak of activity, see below), the surface (measured as the integral of the averaged EMG burst between 0—EMG onset—and 180 ms), and the initial slope (measured as the slope of the linear regression computed between EMG onset and the latency of the maximum value). To get a better and more reliable estimate of the peaks, the averaged bursts were first smoothed, and the latency of the peak was estimated on the filtered data. On the basis of this latency, the amplitude was estimated, on the unfiltered data, as the surface in a small window (±15 ms) centered on the peak latency. This allowed us to avoid spurious detection of invalid peaks, and to reduce the impact of oscillating activities.

### Stepwise logistic regression

Next, in order to determine the simplest model (on the basis of the extracted parameters) that best predicted partial-error detection, eight EMG parameters^2^ were entered in a multivariate logistic regression, using a generalized linear model approach. This procedure allows one to take into account potential correlations between the different parameters (e.g., a burst of longer duration tends to have a higher surface), as well as the possible interaction between the factors, in order to select the simplest combination of parameters that best predicts the dependent variable **in the present case, detection**. The procedure is iterative in the search for the simplest model that accounts adequately for the data. On every iteration, the Bayesian information criterion (BIC), which penalizes model complexity, is computed. The selected model will be the one that minimizes this criterion. The procedure starts with the complete model, including the eight parameters. Then, the stepwise procedure iteratively adds or removes a parameter or an interaction between two parameters from the model (Venables & Ripley, 2002). On every step, the BIC is computed and the quality of the current model is compared to that of the previous estimated ones (Vrieze, 2012). The procedure ends when the minimum BIC is reached. This was done for each participant separately.

This analysis was performed only on detected and undetected categories, to assess the predictive power of each parameter.

## Results

In this “Results” section, we will first present the global performance of the participants on speed and accuracy. We will then present the detection report, irrespective of the actual presence of a partial error, followed by the detection performance of partial errors. In the last part, we will investigate the relationship between the various extracted parameters and detection, and finally evaluate which parameters are the best predictors of detection.

### Behavioral and EMG results

Participants were faster on congruent (359 ms) than on incongruent (391 ms) trials, *t*(17) = 11.00, *p* < .001, and committed more errors on incongruent (7.8 %) than on congruent (2.7 %) trials, *t*(17) = 8.81, *p* < .001, replicating the standard results. They also produced more partial errors on incongruent (18.0 %) than on congruent (7.5 %) trials, *t*(17) = 14.53, *p* < .001. We analyzed other aspects of the performance (notably, the RT distribution and the conditional accuracy function), and all showed the typical pattern reported for this task (data are not shown; see van den Wildenberg et al., 2010, for an overview). Thus, overall, it seems that adding partial-error detection to this task did not affect the performance. This is important, because it indicates that the additional task of evaluating their own performance did not dramatically modify the participants’ strategies.

### Partial-error report

After every trial, participants had to report their detection on a scale ranging from 1 (*sure that they had not produced a partial error*) to 6 (*sure that they had produced a partial error*).

Irrespective of the nature of the trial (partial error or not), participants mainly responded 1 (*no partial error*, 74.9 %). The five consecutive other points in the scale were chosen in 7.4 %, 5.8 %, 2.3 %, 1.3 %, and 3.3 % of trials. They reported “Error” in 5 % of the trials, and were accurate in 94.5 % of such reports. In the following discussion, for the sake of simplicity, categories 1 and 2, 3 and 4, and 5 and 6 will be merged into three categories: “undetected,” “uncertain,” and “detected,” respectively. The proportions of each category, irrespective of the presence or absence of a partial error, were slightly different between congruent and incongruent trials: Participants more often reported “detected” on incongruent trials (3.35 % of such trials) than on congruent trials (1.25 % of the trials; *χ* ^2^ = 263, *p* < .0001).

The same difference was true for the “uncertain” category (5 % vs. 3.15 % for incongruent vs. congruent, respectively; *χ* ^2^ = 161, *p* < .0001). Symmetrically, they reported “undetected” on fewer incongruent trials (38 % vs. 44.3 %; *χ* ^2^ = 363, *p* < .0001). As we will see below, this was due to bias in reporting partial errors, but it does not reflect better detection.

### Partial-error detection

Participants 3 and 7 detected too few partial errors (2 % and 0 %, respectively) for a reliable analysis. Furthermore, Participant 1 never rated trials as “uncertain,” and hence could not be included in the full ANOVA. The remaining analyses hence were performed on 15 participants.^3^ Concerning the “undetected” trials (those rated 1–2) and the “detected” ones (those rated 5–6), reports were highly consistent across participants (see Table 1) and revealed two important facts. First, the average hit ratio was rather low, around 32.3 % (although some variability existed between participants). Second, and importantly, participants’ judgments displayed virtually no false alarms (mean percentage: 1.6 %). Therefore, despite the rather low level of hits, this almost absence of false alarms indicates that responses were not given at random, and hence that the reported partial errors (i.e., hits) were really detected and not lucky guesses.

**Table 1:** Trial repartition, participants’ detection performance, and partial-error (PE) and pure-correct (PC) ratios in the uncertain category

	Trial Repartition			Detection Performance				Uncertain Trials	
Participant	Correct	Errors	Number of PEs	Hits	Misses	False Alarms	Correct Rejections	PE Ratio	PC Ratio
1	95 %	5 %	104	23 %	77 %	0.3 %	99.7 %	–	–
2	96 %	4 %	61	61 %	39 %	2.7 %	97.3 %	8.3 %	88.7 %
3	93 %	7 %	152	2 %	98 %	0.0 %	100.0 %	80.8 %	0.0 %
4	91 %	9 %	241	26 %	74 %	1.0 %	99.0 %	86.6 %	7.5 %
5	89 %	11 %	334	50 %	50 %	1.6 %	98.4 %	79.5 %	9.0 %
6	90 %	10 %	262	29 %	71 %	0.8 %	99.2 %	27.3 %	59.1 %
7^*^	97 %	3 %	45	0 %	100 %	0.1 %	99.9 %	63.6 %	27.3 %
8	91 %	9 %	307	52 %	48 %	0.7 %	99.3 %	89.0 %	8.5 %
9	84 %	15 %	308	10 %	90 %	1.0 %	99.0 %	76.1 %	2.2 %
10	92 %	8 %	197	22 %	78 %	0.6 %	99.4 %	47.5 %	49.2 %
11	95 %	5 %	216	65 %	35 %	1.2 %	98.8 %	25.0 %	66.8 %
12	95 %	5 %	177	42 %	58 %	0.6 %	99.4 %	18.2 %	79.6 %
13	91 %	9 %	394	55 %	45 %	0.0 %	100.0 %	68.7 %	26.4 %
14	87 %	13 %	144	44 %	56 %	8.5 %	91.5 %	12.0 %	83.4 %
15	84 %	16 %	357	21 %	79 %	0.2 %	99.8 %	41.2 %	53.1 %
16	85 %	15 %	200	24 %	76 %	8.0 %	92.0 %	6.8 %	90.8 %
17	84 %	16 %	236	12 %	88 %	0.1 %	99.9 %	32.4 %	59.5 %
18	95 %	5 %	149	43 %	57 %	0.4 %	99.6 %	54.6 %	36.4 %

Participant 1 never reported partial errors as being “uncertain.” For Participant 7 (with the asterisk), only two sessions could be analyzed because of artifacts in the EMG in one session. Given the very low detection of Participants 3 and 7, they were not kept for the second part of the analysis

The hit and false alarm ratios were, however, slightly different between congruent and incongruent trials [hit ratio: 39 % vs. 32 %, for incongruent and congruent, respectively; *t*(14) = 2.69, *p* < .02; false alarm ratio: 2.9 % vs. 0.9 %, for incongruent and congruent trials, respectively; *t*(14) = 4.97, *p* < .001]. Since both measures were affected, we computed *d′*_*e*_,^4^ and found that discriminability did not differ between congruent (*d′*_*e*_ = 1.02) and incongruent (*d′*_*e*_ = 1.09) trials, *t*(14) = 0.6. In contrast, *β* was clearly shifted toward more lenient decisions on incongruent trials (*β* = 2.32) than on congruent ones (*β* = 3.73), *t*(14) = 3.12, *p* < .01. Hence, overall, participants tended to report partial errors more often on incongruent trials (likely because they could infer that their probability was higher on incongruent trials), but did not discriminate partial errors better than on congruent trials.

The “uncertain” category was much less consistent across participants, revealing large variability in the use of this category: Whereas some participants used this category quite often when there was indeed a partial error (e.g., Participant 6 or 9), some others used it mainly for pure-correct trials. This confirms the ambiguous nature of this rating.

Since some of the partial errors could be detected, we searched for the parameters that covaried with detection and, potentially, caused it.

### Relation between partial-error detection and chronometric and physiological indices

To better characterize the differences between detection categories, nine parameters were extracted (see the “Method” section). We conducted ANOVAs on these parameters with Detection (three levels: undetected, uncertain, and detected) and Congruency (two levels) as within-subjects factors. The results are summarized on Table 2. Concerning the congruency effect, already-published results were replicated: Partial errors occurred slightly later for congruent trials, and correction times were shorter for those trials (Burle & Bonnet, 1999; Hasbroucq, Possamaï, Bonnet, & Vidal, 1999). As we have already reported, the other parameters did not differ between the two congruency conditions.

**Table 2:** Results of the full ANOVAs, including detection and congruency for all of the extracted parameters

	Detection					Congruency				Interaction	
	Undetected	Uncertain	Detected	*F*(2, 28)	*p*	Congruent	Incongruent	*F*(1, 14)	*p*	*F*(2, 28)	*p*
IncLat (ms)	208	201	210	0.75	n.s	212	208	5.68	*	0.16	n.s
CT (ms)	127	134	146	31.18	***	134	138	16.00	**	2.81	.
IncSurf (mV)	5.61	6.76	8.52	43.11	***	6.78	7.15	2.93	n.s	5.44	*
IncDur (ms)	23	23	27	37.98	***	25	25	0.11	n.s	4.21	*
IncSlope	42.20	46.30	49.30	16.58	***	45.00	46.90	4.80	n.s	5.62	**
CorSurf (mV)	50.30	50.90	51.80	3.46	*	51.40	50.60	3.40	.	3.01	.
CorDur (ms)	59	60	62	7.22	**	61	61	0.02	n.s	2.16	n.s
CorSlope	85.00	84.40	82.70	1.42	n.s	84.50	83.50	0.27	n.s	0.94	n.s
MT (ms)	96	97	99	12.94	***	97	97	0.08	n.s	3.34	*

n.s.: nonsignificant, .: *p* ≤ 0.1, *p ≤ .05, **p ≤ .01, ***p ≤ .001.

Three main aspects related to detection are worth noting. First, not surprisingly, partial-error “size” (as assessed by IncSurf and IncDur) increased from undetected to detected trials, with “uncertain” trials being in between. Second, correction time (CT) lengthened from undetected to detected partial errors, with uncertain again being in the middle, establishing a relation between the time it takes to correct and the detectability of a partial error. We shall come back on this effect later. Third, undetected, uncertain, and detected partial-error trials also differed in the parameters related to correct response execution (with longer and larger correct EMG bursts and longer motor times for detected than for undetected partial errors, with uncertain being in-between).

**Fig. 2:**
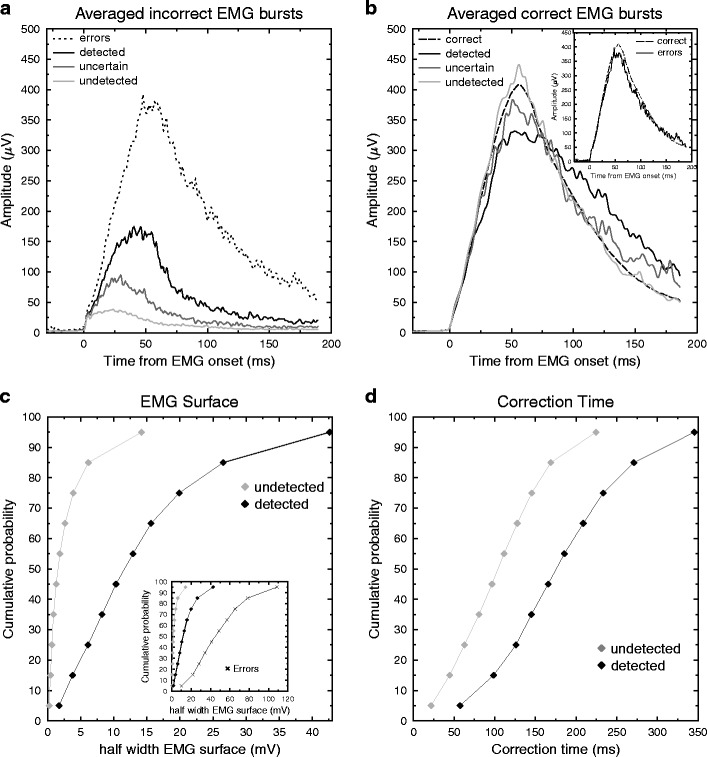
**a** Grand average of the incorrect EMG bursts: The EMG bursts corresponding to partial errors or overt errors were averaged, time-locked to their onsets, for the three detection categories. **b** Grand average of the correct EMG bursts observed on partial-error trials for the three detection categories, and for pure-correct trials. For the sake of visibility, the averaged EMG bursts have been smoothed, but all analyses were performed on the raw, unfiltered signals. (Inset: Grand average of pure-correct and error trials.) **c** Mean cumulative density functions of partial-error surfaces (*IncSurf* ) for undetected (gray diamonds) and detected (black diamonds) partial errors. Although the lowest values of the two distributions are pretty similar, they quickly diverge. (Inset: For the sake of comparison, this graph also shows the cumulative density function of surfaces for overt errors [black crosses].) **d** Mean cumulative density functions of CTs for undetected (gray diamonds) and detected (black diamonds) partial errors. The two distribution shapes are more similar than for those for surfaces, showing a more constant shift.

We then compared the EMG bursts of partial-error trials to the EMG bursts of pure-correct and error trials: The incorrect EMG bursts of partial errors were compared to the error EMG bursts (one-way ANOVA with four modalities of factor detection: detected, uncertain, undetected, and error; Fig. 2a), and the correct EMG bursts of partial-error trials were compared to pure-correct EMG bursts (one-way ANOVA with four modalities of factor detection: detected, uncertain, undetected, and pure-correct; Fig. 2b). Finally, we also compared the pure-correct EMG burst to the one observed on errors. Concerning the latter comparison, as has already been reported (Allain, Carbonnell, Burle, Hasbroucq, & Vidal, 2004), the mean EMG burst has a smaller amplitude on overt errors than on pure-correct responses [*t*(14) = 3.55, *p* < .002], whereas the initial slopes do not differ [*t*(14) = 0.3, *p* = .77; see Fig. 2b, inset]. Comparing the correct EMG bursts for pure-correct and the three categories of partial-error trials revealed clear effects on the initial slope [*F*(3, 42) = 6.72, *p* < .001], on the peak amplitude [*F*(3, 42) = 3.07, *p* < .05], and on MT [*F*(3, 42) = 4.44, *p* < .01]. No main effect on surface was observed [*F*(3, 42) < 1], but inspection of Fig. 2b makes it clear that this was due to a reduced amplitude being compensated for by a wider shape, with the two counteracting each other. Planned orthogonal contrasts revealed that the correct EMG burst of undetected partial errors did not differ from pure-correct trials in any of the significant parameters [slopes, *F*(1, 14) = 2.323, *p* = .15; peak amplitude, *F*(1, 14) < 1; MT, *F*(1, 14) = 1.94, *p* = .185], whereas these two trial types differed from the other two (uncertain and detected), which showed a less steep initial slope [*F*(1, 14) = 12.62, *p* < .005], a decreased peak amplitude [*F*(1, 14) = 9.43, *p* < .005], and a lengthened MT [*F*(1, 14) = 7.02, *p* < .02]. Finally, detected trials produced a longer MT than did uncertain ones [*F*(1, 14) = 10.57, *p* < .01]; these two categories did not differ in either slope [*F*(1, 14) < 1] or surface [*F*(1, 14) = 1.01, *p* = .33]. Concerning the final analysis, comparing the incorrect EMG bursts of partial-error and error trials, Fig. 2a (presenting the mean EMG bursts) shows that even the EMG burst of the largest partial errors (detected ones) differed from the overt-error bursts, with a smaller surface [*t*(14) = 5.16, *p* < .001] and a less steep initial slope [*t*(14) = 4.44, *p* < .001], although a clear overlap exists (Fig. 2c, inset).

Whereas the analyses above provided essential information about the different parameters that covary with detection, they did not allow us to extract which criteria allow the participants to detect their partial errors. In other words, they did not allow us to dissociate the causes from the consequences of detection. Furthermore, the different EMG parameters tend to index similar properties of the EMG bursts and tend to be correlated. Finally, the analyses above did not allow us to test for possible interactions between the factors. We thus performed a stepwise logistic regression to select the most predictive parameters, and their potential interactions. Note that such a logistic regression was restricted to detected and undetected trials, leaving out the uncertain ones. It was thus performed on 16 participants, including Participant 1.

Table 3 presents the combination of parameters that was selected by the stepwise selection for each participant. Two parameters appear to be stable across participants: the incorrect EMG surface, which was selected for 13 of the 16 participants, and the CT, selected for 12 of the 16 participants. These two parameters did not interact for any of the participants, suggesting that they provide independent, and likely complementary, information.^5^ None of the parameters related to correct EMG bursts predicted detection, suggesting that the differences observed on correct EMG bursts are more likely a consequence than a cause of detection.

**Table 3:** Parameters selected by the stepwise selection process for each participant

Participant	IncSurf	CT	IncLat	CT × IncSurf	CorSurf	IncDur × IncSurf	IncDur	IncSlope	IncLat × IncSlope	CorSlope	IncDur × CorSlope
1	***	**				***	*				
2	**	**									
4	***	***			***						
5	***	***	***					***	***		
6	***										
8	***	***		***							
9	***	***	***								
10	***	***									
11		***	***	***							
12	***					***					
13	***	***									
14											
15	***	**									
16	***	***	***								
17	***	***	**	***	***						
18				**			*			*	*
Total	13	12	5	4	2	2	1	1	1	1	1

*p < .05, **p < .01, ***p < .001.

### Predictive values of the selected parameters

**Fig. 3:**
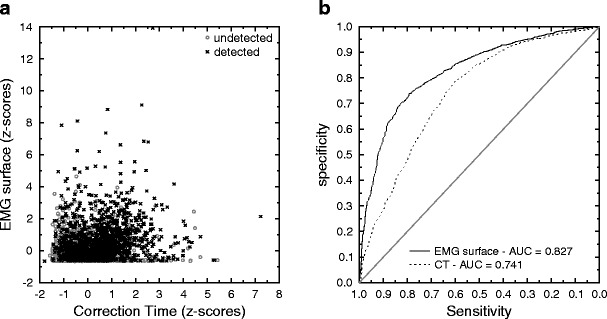
**a** Scatterplot of IncSurf as a function of CT for all participants (after *z*-score computation). The overlap between undetected and detected partial errors is large, and it is clear that neither of the two parameters in itself allows for a clear prediction of partial-error detection. It can be noted, however, that above a virtual decreasing diagonal, most of the points belong to the detected class, confirming that the combination of the two parameters is necessary for classifying the trials. **b** Receiver operating characteristic curves for EMG surface (solid line) and CT (dashed line). The area under the curve (AUC) is larger for EMG surface than for CT

Although the two selected parameters differed significantly between detected and undetected partial errors, this difference was true on average, but the dissociation was far less clear at the individual-trial level. Indeed, Fig. 2 (panels c and d) shows the cumulative density functions (CDFs) of the EMG surface (panel c) for undetected and detected partial errors, along with the CDF for the overt-error surface, as an inset. Panel d shows the CDFs of CT for undetected and detected partial errors. As can clearly be seen, both parameters have large overlaps in their distributions of detected and undetected partial errors. The overlap is also evident on Fig. 3a, which presents the scatterplot of the EMG surface as a function of CT for all of the participants (*z*-score transform) for detected and undetected partial errors. To better characterize the predictive values of these two parameters, we first computed the receiver operating characteristic (ROC) curves and the corresponding areas under the curve (AUCs) for both parameters using the pROC library (Robin et al., 2011) under the R environment (R Development Core Team, 2012), on the pooled data across participants (Fig. 3b). Second, we computed the corresponding regression coefficients on the *z*-score values for the EMG surface and CT. The two AUCs were significantly different from 0.5 [as assessed by one-tailed *t* tests: 0.863 for EMG surface, *t*(15) = 15.0, *p* < .001, and 0.742 for CT, *t*(15) = 7.7, *p* < .001], indicating that the two parameters have clear discriminative power. However, this discriminative power also differs between the two parameters.

Indeed, as is indicated by Fig. 3b, the AUC for EMG surface is higher than that for CT, which is confirmed by a clear significant difference (*Z* = −8.08, *p* < .001) as assessed by DeLong’s test (DeLong, DeLong, & Clarke-Pearson, 1988). Additionally, the regression coefficient obtained for EMG surface (*β*_IncSurf_ = 4.31) was higher than the one obtained for CT (*β*_CT_ = 1.31), *t*(15) = 2.78, *p* < .01. These two results suggest that incorrect EMG surface is more efficient than CT in predicting partial-error detection, despite the fact that the information provided by both parameters seems to be essential, and complementary, for partial-error detection.

## Discussion

In the 1960s, Rabbitt and colleagues (Rabbitt, 1966; Rabbitt & Vyas, 1981) established that participants can correct most of their errors, although conscious correction reports are fewer. The use of electrophysiological markers has shown, however, that erring is not an all-or-none process, but is instead a gradual one. In this regard, the recording of muscular activity of the muscles involved in response execution is essential. EMG recordings have revealed that a subthreshold response activation can be observed on the muscles involved in the incorrect response in about 15 % of the behaviorally correct trials. Such “partial errors” have been shown to induce a brain response initially reported for overt errors (Burle et al., 2008; Masaki & Segalowitz, 2004; Scheffers, Coles, Bernstein, Gehring, & Donchin, 1996; Vidal et al., 2000), called the “error negativity” (Falkenstein et al., 1991) or “error-related negativity” (Gehring et al., 1993). Here, we investigated whether partial errors are consciously detected by the participants, and if so, which features allow their detection, or at least covary with it.

The detection results were highly coherent across participants, showing that most partial errors remained consciously undetected. Indeed less than one third of the partial errors were reported. Importantly, and also consistently, participants reported virtually no false alarms, indicating that responses were not given at random, and that when participants did report a partial error, it was not a lucky guess. In summary, participants could detect only a few of their partial errors, but with high reliability. Another aspect that deserves comment is the absence of a congruency effect on the detection performance. Indeed, although participants tended to report the presence of a partial error more often on incongruent trials, their true detection was comparable for congruent and incongruent trials (no difference in *d′*_*e*_), despite the fact that partial errors were likely triggered by different processes in these two types of trials (partial errors on incongruent trials are largely triggered by stimulus position, whereas most of them are likely guesses on congruent trials; Gratton et al., 1992).

In a second step, we searched for the chronometric and physiological predictors of partial-error detection. Two parameters reliably emerged across participants: incorrect EMG surface and correction time. We will now discuss these two parameters.

The first, and most reliable and predictive, feature was the size (indexed by various parameters) of the incorrect EMG burst: The “larger” the incorrect EMG burst, the more likely was its detection. Such a relationship was certainly not unexpected, since one intuitively thinks that the “stronger” the incorrect response activation, the more likely that one can “feel” it. Although this seems intuitive, scratching below the surface makes the link less straightforward than it may seem. From a physiological point of view, how could the EMG burst amplitude be related to its detection? EMG activity detection could rely on the strength of the reafferent proprioceptive signal triggered by the muscle contraction, since larger contractions should induce stronger reafferences. Although it is possible, this option seems unlikely: First, a study on a completely deafferented patient has suggested that reafferences play little role in partial-error production and correction (Allain, Hasbroucq, Burle, Grapperon, & Vidal, 2004). Furthermore, proprioceptive afferences are gated during motor command sending (Abbruzzese, Ratto, Favale, & Abbruzzese, 1981), and since partial errors are very small muscular activations, without a clear motor twitch, reafferences reaching the cortex are probably extremely weak, making this an unlikely signal for conscious detection.

Alternatively, detection could be based on the strength of the central motor command. As a matter of fact, partial errors index (in)voluntary motor commands, and clear primary motor cortex (M1) activation can be observed before and during the partial-error burst (Burle et al., 2008, Fig. 8). Given the monotonic link between movement strength and primary motor cortex activity (Evarts, 1968), one expects to find larger M1 activation for detected than for undetected partial errors. Sending a motor command also triggers an efference copy (Angel & Malenka, 1972; Wolpert, 1997), which is thought to be a mere copy of the sent command, allowing for comparison between the intended and the actually performed movement. The medial part of the frontal cortex (supplementary motor area and/or anterior cingulate cortex) has been shown to be involved both in the generation of such efferent signals (Haggard & Magno, 1999; Haggard & Whitford, 2004) and in evaluating actions (Carter et al., 1998; Coles, Scheffers, & Holroyd, 2001; Falkenstein et al., 1991; Roger, Bénar, Vidal, Hasbroucq, & Burle, 2010). As a matter of fact, it has been proposed that action monitoring could be based on such efference copies (Allain, Hasbroucq, et al., 2004; Coles et al., 2001; Gehring et al., 1993). In this case, the efference copy could be at the origin of the detection: If its strength is too weak (due to a weak motor command), it may not access consciousness, whereas a larger one would. Although this is admittedly speculative, such a proposal calls for cortical investigations of partial-error detection.

Correction time was also longer for detected than for undetected partial errors, and this difference allowed us to classify these two categories of trials fairly well. Importantly, since its contribution to classification is independent from that of the EMG surface, it is not a mere consequence of the incorrect EMG burst size. The relation between CT duration and detection echoes a recent proposal treating error detection as a decision-making mechanism (Steinhauser & Yeung, 2010). Standard models of decision making assume that one accumulates evidence in favor of the various alternatives until enough evidence has been accumulated for the decision to be taken (Ratcliff & McKoon, 2008). In this framework, the more time that one has to accumulate evidence, the more accurate the decision. Consistent with this view, Rabbitt (2002) reported that error commission takes time to reach conscious access. He also showed that an interfering stimulus occurring too early after an error prevents conscious access. In the present context, the correction time data allow an integration of the two views: After a partial error, an accumulation process starts. One may speculate that the speed of accumulation (the drift rate, in the standard diffusion model) could be related to the strength of the efference copy, with larger partial errors inducing faster accumulation. If the correct response occurred too early, it will interfere with, and hence interrupt, the accumulation process, preventing it from reaching the threshold necessary for conscious detection. In contrast, if the corrective response occurred later, the accumulation process would have enough time to reach the threshold, and the partial error would be detected.

The correct(ive) EMG burst leading to an overt response following a partial error was also affected by detection (larger and longer for detected than for undetected partial errors). It was also somewhat less efficient, as revealed by the increased motor time. Interestingly, contrary to the incorrect EMG size and correction time, its parameters do not allow for prediction of the detection outcome, suggesting that it is likely a consequence, rather than a cause, of detection. This view is also supported by a comparison with pure-correct bursts: When a partial error was detected, the corrective EMG activity significantly differed from that in pure-correct trials, whereas undetected partial errors produced corrective EMG that was not different from pure-correct bursts. Interestingly, corrective EMG sizes for uncertain partial-error detection were in between the detected and undetected values, but were somewhat closer to the detected ones.

A last comment is in order: at the cerebral level, it has been shown that partial errors evoke an Ne, but a later positive component, the error positivity (Pe), is not observed on such trials (Burle et al., 2008; Vidal et al., 2000). Since the Pe seems specific to detected errors (Endrass et al., 2007; Murphy, Robertson, Allen, Hester, & O’Connell, 2012; Nieuwenhuis et al., 2001; O’Connell et al., 2007), its absence on partial-error trials is consistent with the present results showing that most partial errors did not access the conscious level. However, the presence of an Ne, along with behavioral adjustments such as post-(partial)-error slowing (Allain et al., 2009), indicates that, even without conscious access, partial errors are detected by the nervous system, which in turn triggers control mechanisms. Note that, at least for saccadic eye movements, detected and undetected erroneous eye movements generate Ne’s of the same amplitude (Endrass et al., 2007; Nieuwenhuis et al., 2001; O’Connell et al., 2007), suggesting that this wave is not related to conscious detection. Whether this would also hold for partial erroneous limb-response activation is still an open question.

In summary, most partial errors remain perfectly undetected, confirming that corrective behavior can occur without conscious access. When they are detected, the strength of the motor command triggering the incorrect EMG burst might be an essential signal for detection, likely through the efference copy sent to the control centers, especially within the medial wall. Second, conscious access takes time to build up, and if the corrective response appears too early with respect to the partial error, it has a masking effect, preventing conscious access. Finally, it seems that having detected the partial error modifies the corrective behavior, leading to an enlarged, although less efficient (as assessed by motor time), corrective motor command. These facts being established, one can now search for the cortical determinants of conscious (partial) error detection. All together, these results suggest that conscious access is not a necessary condition for the recruitment of executive control processes.

## Footnotes

1 Indeed, for incorrect EMGs, the standard deviations of both IncSurf and IncDur (10.6 and 29.8, respectively) are less than the raw surface and raw EMG durations (16.4 and 56.5, respectively). Furthermore, IncSurf and IncDur are highly correlated to the raw surface and duration estimates.

2 For unclear reasons, adding MT to the model prevented the algorithm from converging for several participants. This factor was thus excluded from the analysis, since we did not expect it to be a predictor, given the absence of prediction of the correct EMG burst parameters (see below).

3 A full ANOVA restricted to “undetected” and “detected” trials was performed on the 16 participants, including Participant 1. This ANOVA led to the same conclusions.

4 Since we verified that variance equalities of the noise and signal distributions were not ensured, we used a corrected measure of *d′*, named *d′*_*e*_ (Gescheider, 1976).

5 To assess the validity of the regression, the variance inflation factor (VIF; see Kutner, Nachtsheim, & Neter, 2004) was computed. The mean VIF was found to be 1.698, and only two values (out of 128) were above 5 (5.4 and 5.27), whereas an upper limit of 10 is classically prescribed.
